# Rearing Children

**Published:** 1860-10

**Authors:** 


### REARING CHILDREN.

1. CHILDREN should not go to school until six years old.

2. Should not learn at home during that time more than the alphabet, religious teachings excepted.

3. Should be fed with plain substantial food, at regular intervals of not less than four hours.

4. Should not be allowed to eat any thing within two hours of bed-time.

5. Should have nothing for supper but a single cup of warm drink, such as very weak tea of some kind, or cambric tea or warm milk and water, with one slice of cold bread and butter—nothing else.

6. Should sleep in separate beds, on hair-mattresses, without caps, feet first well warmed by the fire or rubbed with the hands until perfectly dry; extra covering on the lower limbs, but little on the body.

7. Should be compelled to be out of doors for the greater part of daylight, from after breakfast until half an hour before sun-down, unless in damp, raw weather, when they should not be allowed to go outside the door.

8. Never limit a healthy child as to sleeping or eating, except at supper; but compel regularity as to both; it is of great importance.

9. Never compel a child to sit still, nor interfere with its enjoyment, as long as it is not actually injurious to person or property, or against good morals.

10. Never threaten a child: it is cruel, unjust and dangerous. What you have to do, do it, and be done with it.

11. Never speak harshly or angrily, but mildly, kindly, and, when really needed, firmly—no more.

12. By all means arrange it so that the last words between you and your children at bed-time, especially the younger ones, shall be words of unmixed lovingness and affection.

